# Acetabular Morphotype Is Associated with Change in Acetabular Orientation Between Supine and Standing

**DOI:** 10.2106/JBJS.OA.26.00151

**Published:** 2026-07-13

**Authors:** Camille Vorimore, Jeroen C.F. Verhaegen, Chuan Kong, Eduardo Viglietta, Paul Beaulé, George Grammatopoulos

**Affiliations:** 1 Department of Orthopaedic Surgery, The Ottawa Hospital, Ottawa, Ontario, Canada; 2 Cochin Hospital, Paris, France; 3 Department of Orthopaedic Surgery, University Hospital Antwerp, Edegem, Belgium; 4 Orthopaedic Centre Antwerp, AZ Monica, Antwerp, Belgium; 5 Auckland City Hospital, Auckland, New Zealand; 6 Department of Orthopaedic Surgery, La Sapienza University, Roma, Italy

## Abstract

**Background:**

Debate exists on which functional position (supine or standing) should form the basis of radiographic assessment of the native acetabulum. Pelvic tilt (PT) differs between positions influencing radiographic acetabular assessment. This study aims to (1) quantify PT changes from supine to standing amongst volunteers and patients; (2) assess changes in acetabular parameters between the postures; and (3) determine whether changes in acetabular parameters differ according to acetabular morphotype.

**Methods:**

This is a prospective, consecutive, cohort study of 105 asymptomatic volunteers (53% male; mean age 36.5 ± 13.6 years; body mass index [BMI] 25.0 ± 2.0 kg/m^2^) and 437 patients presenting to a hip preservation clinic without osteoarthritis (Tonnis ≤1) (40% male; mean age 35.7 ± 8.6 years; BMI 25.0 ± 2.0 kg/m^2^). All underwent standardized supine and standing anteroposterior pelvic radiographs. PT change was quantified using the sacrofemoral-pubic (SFP) angle. Acetabular morphology was evaluated in detail to characterize morphotype according to the Ottawa classification (lateral dysplasia, anterior dysplasia, posterior dysplasia), pincer or “normal.” Changes between supine and standing were compared between cohorts and morphotypes.

**Results:**

The mean SFP decreased by −3.7° (IC95% 4.0-3.5) from supine to standing. Acetabular measurements demonstrated no differences in change between volunteers and symptomatic patients when transitioning between supine to standing. Measurements of lateral coverage changed minimally between supine (volunteers: 30.7° ± 6.1°; symptomatics: 27.3° ± 8.3°) and standing (volunteers: 30.3° ± 5.7°; symptomatics: 26.1° ± 8.1°), whereas measurements reflecting anteroposterior coverage changed significantly (ΔAWI volunteers: −0.10 ± 0.08 and symptomatics: −0.09 ± 0.07; p < 0.001 and ΔPWI volunteers: 0.09 ± 0.08 and symptomatics: 0.09 ± 0.07; p < 0.001). Patients with anterior dysplasia demonstrated smaller ΔSFP (−1.5° ± 2.5° vs. −3.7° ± 2.9°; p = 0.004) compared with the rest of the cohort. By contrast, posterior dysplasia showed greater ΔSFP (−4.5° ± 2.7° vs. −3.6° ± 2.9°; p = 0.128) than the rest.

**Conclusions:**

Groups demonstrated comparable changes in PT and acetabular parameters between positions. The magnitude and direction of change was associated with acetabular morphology uncoupling compensatory mechanisms. Standing radiographs may be preferred to supine, as they account for compensatory mechanisms.

**Level of Evidence:**

Level II, diagnostic. See Instructions for Authors for a complete description of levels of evidence.

## Introduction

Radiographic evaluation of the acetabulum is an important part of diagnostic and treatment algorithms for patients presenting with hip pain^1-6^. Assessment of acetabular morphology is based on multiple parameters, including lateral center-edge angle (LCEA) and acetabular index (AI) for lateral coverage, and anterior (AWI) and posterior wall indices (PWI) for antero-posterior coverage^4,7,8^.

Historic, normative, values have been described on supine, anteroposterior (AP) pelvic radiographs. However, supine radiographs may not accurately represent acetabular orientation in a functional, standing position, where joint stability is more relevant due to load-bearing and risk of instability^9-13^. Pelvic tilt (PT) changes during postural transitions lead to differences in acetabular appearance and femoral head coverage^14,15^. These positional changes may be influenced by the underlying acetabular morphology, as a compensatory mechanism for pathomechanics, associated with acetabular undercoverage or overcoverage.

This study aims to (1) quantify PT changes from supine to standing among asymptomatic volunteers and symptomatic hip patients, (2) assess changes in acetabular parameters between postures in both cohorts, and (3) determine whether changes in acetabular parameters differ according to distinct acetabular morphotypes.

## Methods

### Study Design

**Fig. 1:**
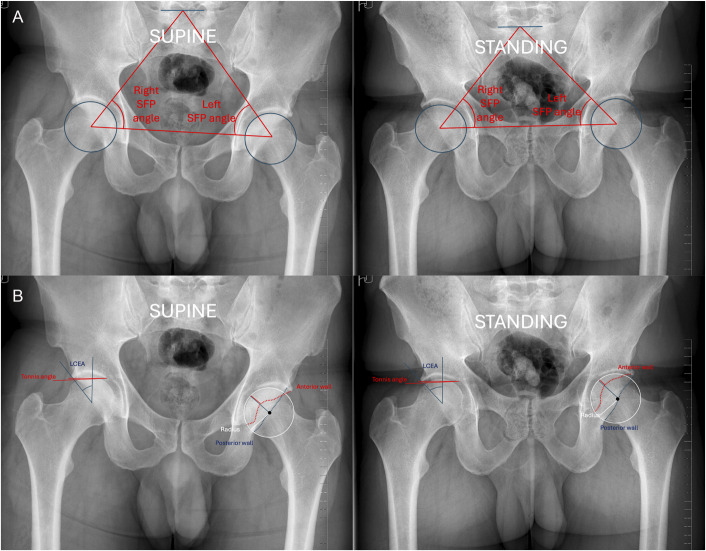
**Fig. 1-A** AP pelvic radiograph in supine and (**Fig. 1-B**) standing position illustrating the measurements performed in this study. AWI = anterior wall index, LCEA = lateral center-edge angle, PWI = posterior wall index, and SFP angle = sacro-femoro-pubic angle.

This was an REB-approved (REB No.: 20210082-01H), prospective, consecutive, single-center, case-control study conducted at a single academic tertiary referral center with a large hip preservation practice (The Ottawa Hospital, Canada). Two cohorts were recruited between 2021 and 2023: (1) asymptomatic volunteers, including medical students, hospital staff, and patients attending fracture clinics for upper limb injuries and (2) symptomatic patients presenting with hip pain (Fig. 1).

An a priori power calculation was performed to evaluate differences in PT between supine and standing positions in dysplastic and retroverted patients. This calculation was based on findings from previous studies, Jenkinson et al.^16^ reported a PT change of 7.6° ± 4.5° in patients with retroversion and Tachibana et al.^11^ reported a PT change of 6.0° in dysplastic patients. Thus, a minimum of 336 symptomatic hips was determined to necessary to achieve adequate statistical power (1-β = 0.90; α = 0.05).

### Cohort Description

Inclusion criteria for both cohorts were age over 18 years-old, absence of osteoarthritis (Tonnis Grade ≤1), no prior hip or spine pathology or surgery. In addition, the asymptomatic cohort required the absence of hip symptoms (Oxford Hip Score > 45). All participants provided informed consent. A total of 110 volunteers were recruited between May 1, 2021, and January 31, 2023, excluding those without adequate quality of standing radiographs (n = 3) and those with evidence of spondylolisthesis (n = 1) or scoliosis (n = 1). In addition, 448 symptomatic patients were recruited during the same period, excluding those without adequate quality of standing radiographs (n = 11).

Of the participants, 53% (56/105) of the volunteers and 40% (176/437) of the symptomatic patients were male (p = 0.012). The mean age ± SD was 36.5 ± 13.6 years for volunteers and 35.7 ± 8.6 years for symptomatic patients (p = 0.447), while the mean BMI was 25.0 ± 2.0 kg/m^2^ for volunteers and 27.2 ± 6.3 kg/m^2^ for symptomatic patients (p = 0.307) (Table I). In the symptomatic cohort, indications included hip dysplasia in 98 hips (23%), Femoro-Acetabular Impingement in 226 hips (53%), mixed pathology in 42 hips (10%), isolated labral tear in 31 hips (7%), and other indications in 32 hips (7%).

**TABLE I:** Descriptive Data

	Volunteers	Symptomatic		p
Age (y ± SD [range])	36.5 ± 13.6 (23-77)	35.7 ± 8.6 (16-58)		0.447
BMI (kg/m^2^ ± SD [range])	25.0 ± 2.0 (22-28)	27.2 ± 6.3 (17-35)		0.307
Side, right/left (%right)	51/54 (49%)	224/203 (52%)		0.318
Sex, male/female (%male)	56/49 (53%)	171/248 (41%)		0.012
Diagnosis, n (%)		Global dysplasia	74 (18%)	
		Anterior dysplasia	14 (3%)	
		Posterior dysplasia	28 (7%)	
		FAI-Pincer	24 (6%)	
		FAI-CAM	79 (19%)	
		Combined FAI	86 (21%)	
		Mixed FAI-dysplasia	14 (3%)	
		Labral tear	30 (7%)	
		Others	70 (17%)	
		Total	419 (100%)	

BMI = body mass index, FAI = femoroacetabular impingement, and Others = combined morphologies with symptoms considered to arise predominantly from hip joint layers 3 and 4, managed nonoperatively^1^.

p-values represent comparisons of descriptive data between volunteers and symptomatic patients.

### Radiographic Assessments

All participants underwent radiographic assessment including supine and standing AP radiographs of the pelvis, in a standardized fashion^17^, consistent with current standards and guidelines for medical imaging and in accordance with institutional review board recommendations^18^. The x-ray beam was intentionally not corrected to homogenize the coccyx-symphyseal distance^19^ for any of the patients because the native variation in pelvic morphology and tilt is of interest in our clinical algorithm. For the volunteers, one side was randomly selected for radiographic assessment, resulting in 51 right hips and 54 left hips.

Each hip was evaluated based on radiographic parameters, including lateral coverage (LCEA^20^ and AI^21^), anterior and posterior coverage (AWI^22^ and PWI^22^), and indicators of retroversion (posterior wall sign [PWS]^23^, crossover sign [COS]^23^, and ischial spine sign [ISS]^24^). Acetabular morphology was evaluated in detail to characterize morphotype according to the Ottawa classification (lateral/global dysplasia, anterior dysplasia, posterior dysplasia), pincer (LCEA > 40°) or “normal” (Table II)^7,25^.

**TABLE II:** Changes in Radiographic Acetabular Measurements Between Supine and Standing Radiographs in Volunteers and Symptomatic Patients

	Supine			Standing			ΔSupine Standing				
	Volunteers (n = 105)	Symptomatics (n = 419)	p*	Volunteers (n = 105)	Symptomatics (n = 419)	p*	Volunteers (n = 105)	p**	Symptomatics (n = 419)	p**	p*
LCEA (°)	30.7 ± 6.1	27.3 ± 8.3	<0.001	30.3 ± 5.7	26.1 ± 8.1	<0.001	−0.40 ± 2.0	0.042	−1.22 ± 2.6	<0.001	0.003
Tonnis angle (°)	6.3 ± 4.4	2.6 ± 6.9	<0.001	7.4 ± 4.8	3.4 ± 6.8	<0.001	1.05 ± 2.5	<0.001	0.77 ± 2.2	<0.001	0.272
COS (n, %)	36 (38)	164 (39)	0.471	21 (20)	81 (19)	0.810	−15 (14)	<0.001	−84 (20)	<0.001	0.348
COR	0.28 ± 0.11	0.25 ± 0.11	0.109	0.25 ± 0.10	0.19 ± 0.11	0.023	−0.14 ± 0.13	0.003	−0.14 ± 0.13	<0.001	0.958
COR >0.3 (n, %)	12 (11)	41 (10)	0.565	6 (6)	14 (3)	0.236	−6 (6)	<0.001	−27 (6)	<0.001	0.944
ISS (n, %)	36 (34)	96 (23)	0.010	16 (15)	24 (6)	<0.001	−20 (19)	<0.001	−72 (17)	<0.001	0.437
PWS (n, %)	70 (67)	214 (51)	0.002	40 (38)	142 (34)	0.333	−30 (29)	<0.001	−72 (17)	<0.001	0.106
AWI	0.49 ± 0.13	0.46 ± 0.14	0.050	0.39 ± 0.14	0.37 ± 0.13	0.109	−0.10 ± 0.08	<0.001	−0.09 ± 0.07	<0.001	0.460
PWI	0.94 ± 0.15	0.95 ± 0.16	0.356	1.03 ± 0.15	1.04 ± 0.16	0.510	0.09 ± 0.08	<0.001	0.09 ± 0.07	<0.001	0.526
AWI/PWI ratio	0.54 ± 0.21	0.50 ± 0.17	0.012	0.40 ± 0.17	0.36 ± 0.14	0.041	0.15 ± 0.12	<0.001	0.13 ± 0.11	<0.001	0.206
SFP (°)							−4.05 ± 3.58	<0.001	−3.7 ± 3.07	<0.001	0.232
Lateral/global dysplasia (n, %)	6 (6)	74 (18)	0.003	7 (7)	92 (22)	<0.001	1 (1)	0.007	18 (4)	<0.001	0.101
Anterior dysplasia (n, %)	7 (7)	14 (3)	0.108	19 (18)	43 (10)	0.021	12 (11)	0.005	29 (7)	<0.001	0.124
Posterior dysplasia (n, %)	9 (9)	28 (7)	0.460	4 (4)	4 (1)	0.030	−5 (5)	<0.001	−24 (6)	<0.001	0.699
Pincer-FAI (n, %)	8 (8)	29 (7)	0.577	6 (6)	17 (4)	0.231	−2 (2)	<0.001	−12 (3)	<0.001	0.586
Others (n, %)	75 (71)	284 (68)	0.171	69 (66)	273 (65)	0.136	−6 (6)	0.001	−11 (3)	<0.001	0.110

AWI = anterior wall index, COS = crossover sign, COR = crossover ratio, FAI = femoroacetabular impingement, ISS = ischial spine sign, LCEA = lateral center-edge angle, PWS = posterior wall sign, PWI = posterior wall index, and SFP = sacro-femoro-pubic angle.

* p-value comparison of radiographic parameters between volunteers and symptomatic patients.

** p-value comparison of radiographic parameters between supine and standing position.

Differences in acetabular parameters (Δ) between the supine and standing positions were analyzed within each patient and stratified according to acetabular morphotype. Clinically important differences between supine and standing position were defined as a difference exceeding 3° for angular measurements and greater than 0.03 for AWIs, based on studies from Troelsen et al.^26^ and Anderson et al.^27^. These thresholds were defined based on the values obtained for LCEA and AWI in both studies (using mean change between position ± 2 × SDs).

The sacrofemoral‐pubic angle (SFP) was determined from AP pelvic radiographs^28^. PT change between supine and standing positions was determined by the difference in the SFP (ΔSFP = SFPsupine − SFPstanding)^28-31^. ΔSFP has been shown to be a reliable parameter to assess the difference in PT in different positions for a given patient^30^. An increase in PT corresponds to a posterior rotation of the pelvis, corresponding to a decrease in SFP, whereas a decrease in PT reflects an anterior rotation of the pelvis and an increase in SFP (Table III).

**TABLE III:** Changes in Acetabular Measurements Between Supine and Standing Positions According to Acetabular Parameters and Diagnosis in Symptomatic Patients

	Number n (%)	Acetabular Change Between Supine and Standing Position					
		ΔAWI	p	ΔPWI	p	ΔSFP	p
Groups according to acetabular morphology							
LCEA > 25°	271 (65)	−0.098	**0.008**	0.086	0.697	−3.743	0.372
LCEA < 25°	148 (35)	−0.078		0.084		−3.480	
Tonnis angle < 10°	374 (89)	−0.092	0.217	0.084	0.348	−3.608	0.426
Tonnis angle > 10°	45 (11)	−0.078		0.094		−3.958	
No COS	261 (62)	−0.082	**0.006**	0.075	**<0.001**	−3.457	0.088
COS	158 (38)	−0.103		0.102		−4.000	
No ISS	329 (79)	−0.085	**0.003**	0.079	**<0.001**	−3.458	**0.011**
ISS	90 (21)	−0.110		0.107		−4.319	
AWI < 0.31	52 (12)	−0.042	**<0.001**	0.066	**0.049**	−2.784	**0.031**
0.31 < AWI < 0.51	224 (53)	−0.085		0.085		−3.634	
AWI > 0.51	143 (34)	−0.118		0.093		−4.000	
PWI < 0.81	73 (17)	−0.102	0.208	0.114	**<0.001**	−4.087	0.303
0.81 < PWI < 1.14	299 (71)	−0.090		0.082		−3.592	
PWI >1.14	47 (11)	−0.077		0.057		−3.326	
AWI/PWI > 0.6	330 (79)	−0.081	**<0.001**	0.080	**0.003**	−3.512	0.066
AWI/PWI < 0.6	89 (21)	−0.125		0.105		−4.138	
Diagnosis							
Global dysplasia	74 (18)	−0.075	**0.048**	0.089	0.591	−3.603	0.883
Anterior dysplasia	14 (3)	−0.031	**0.002**	0.026	**0.001**	−1.471	**0.004**
Posterior dysplasia	28 (7)	−0.120	**0.029**	0.127	**0.001**	−4.461	0.128
FAI-Pincer	24 (6)	−0.116	0.086	0.104	0.189	−4.054	0.736
FAI-CAM	79 (19)	−0.086	0.540	0.082	0.578	−3.578	0.812
Combined FAI	86 (21)	−0.097	0.351	0.074	0.107	−3.372	0.324
Mixed FAI-dysplasia	14 (3)	−0.079	0.541	0.068	0.361	−2.236	0.065
Labral tear	30 (7)	−0.099	0.514	0.085	0.999	−4.268	0.229
Others	70 (17)	−0.092	0.845	0.092	0.363	−4.078	0.155
Total	**419 (100)**	−0.091		0.085		−3.649	

AWI = anterior wall index, COS = crossover sign, FAI = femoroacetabular impingement, ISS = ischial spine sign, LCEA = lateral center-edge angle, PWI = posterior wall index, and SFP = sacro-femoro-pubic angle.

p-values were calculated by comparing each diagnostic subgroup with the remainder of the cohort.

All radiographic measurements were performed by 3 hip preservation research fellows (C.V., C.K., E.V.) using Picture Archiving Communication System software for digital radiographs. Measurements were repeated for 15% of randomly selected datasets in a blinded fashion to evaluate for intra-observer reliability. The senior author (G.G.), a hip preservation fellowship-trained orthopaedic surgeon, and one of the authors (C.V.) repeated measurements in a blinded method for 15% of randomly selected datasets to assess for inter-observer reliability. Interobserver and intraobserver reliabilities were calculated using the correlation coefficient with a 2-way mixed model and showed excellent agreement of between 0.742 and 1 (Supplemental Table I).

### Statistical Analysis

Means and change of acetabular parameter measurements between postures were compared with paired *T*-tests. To compare continuous variables between independent groups, a Mann-Whitney *U* test was used. The χ^2^ exact test was used to test for differences between categorical variables. To assess the variability of PT and acetabular parameters within the cohort, the variability was calculated, defined as 2 × SD. Statistical analysis was performed using SPSS v25 (SPSS). A p-value < 0.05 was considered significant.

**Fig. 2:**
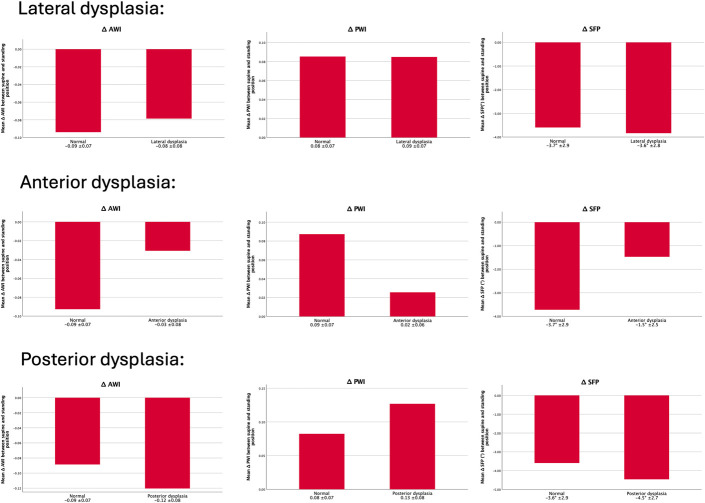
Comparison of changes in anterior wall index (ΔAWI), posterior wall index (ΔPWI), and sacrofemoral-pubic angle (ΔSFP) between patients with lateral dysplasia, anterior dysplasia, posterior dysplasia, and the remainder of the cohort when transitioning from the supine to the standing position.

**TABLE IV:** Acetabular Groups According to Supine and Standing Position

	Volunteers (n = 105)				Symptomatics (n = 429)			
Groups	Supine	Standing	ΔSupine-Standing	p	Supine	Standing	ΔSupine-Standing	p
Lateral/global dysplasia	6 (6%)	7 (7%)	1 (1%)	0.007	74 (17%)	92 (21%)	18 (4%)	<0.001
Anterior dysplasia	7 (7%)	19 (18%)	12 (11%)	0.005	14 (3%)	43 (10%)	29 (7%)	<0.001
Posterior dysplasia	9 (9%)	4 (4%)	−5 (5%)	<0.001	28 (7%)	4 (1%)	−24 (6%)	<0.001
Pincer-FAI	8 (8%)	6 (6%)	−2 (2%)	<0.001	29 (7%)	17 (4%)	−12 (3%)	<0.001
Others	75 (71%)	69 (66%)	−6 (6%)	0.001	284 (66%)	273 (64%)	−11 (3%)	<0.001

FAI = femoroacetabular impingement.

p-value: comparison of prevalence of acetabular groups between supine and standing position.

**TABLE V:** Changes in Radiographic Acetabular Measurements According to Acetabular Parameters Between Supine and Standing Radiographs in Symptomatic Patients

	Number n (%)	Acetabular Change Between Supine and Standing Position					
Groups According to Acetabular Morphology		ΔAWI	p	ΔPWI	p	ΔSFP	p
LCEA > 25°	276 (64)	−0.098	**0.008**	0.086	0.697	−3.743	0.372
LCEA < 25°	153 (36)	−0.078		0.084		−3.480	
AI < 10°	379 (88)	−0.092	0.217	0.084	0.348	−3.608	0.426
AI > 10°	50 (12)	−0.078		0.094		−3.958	
No COS	265 (62)	−0.082	**0.006**	0.075	**<0.001**	−3.457	0.088
COS	163 (38)	−0.103		0.102		−4.000	
No ISS	334 (78)	−0.085	**0.003**	0.079	**<0.001**	−3.458	**0.011**
ISS	95 (22)	−0.110		0.107		−4.319	
AWI < 0.31	55 (13)	−0.042	**<0.001**	0.066	**0.049**	−2.784	**0.031**
0.31 < AWI < 0.51	228 (53)	−0.085		0.085		−3.634	
AWI > 0.51	146 (34)	−0.118		0.093		−4.000	
PWI < 0.81	75 (17)	−0.102	0.208	0.114	**<0.001**	−4.087	0.303
0.81 < PWI < 1.14	306 (71)	−0.090		0.082		−3.592	
PWI > 1.14	48 (11)	−0.077		0.057		−3.326	
AWI/PWI > 0.6	335 (78)	−0.081	**<0.001**	0.080	**0.003**	−3.512	0.066
AWI/PWI < 0.6	94 (22)	−0.125		0.105		−4.138	

Bold values indicate statistical significance (P < 0.05). AI = acetabular index, AWI = anterior wall index, FAI = femoroacetabular impingement, ISS = ischial spine sign, LCEA = lateral center-edge angle, PWI = posterior wall index, and SFP = sacro-femoro-pubic angle.

**TABLE VI:** Multivariate Analysis of Variables Associated with Changes in Absolute ΔSFP

	Unstandardized β Coefficients	Standard Error	Standardized β Coefficients	p
Constant	5.807	0.624		<0.001
Age	−0.018	0.012	−0.067	0.123
Gender	−0.528	0.299	−0.100	**0.022**
Lateral dysplasia	−0.138	0.322	−0.019	0.668
Anterior dysplasia	−1.446	0.579	−0.108	**0.013**
Posterior dysplasia	1.159	0.450	0.113	**0.010**
Pincer-FAI	0.443	0.439	0.044	0.313
Hip symptoms	0.498	0.286	−0.076	0.082

FAI = femoroacetabular impingement and SFP = sacro-femoro-pubic angle.

## Results

1. Pelvic change between supine and standing
  The mean SFP decreased (i.e., PT increased) by 3.7° (95% CI 4.0-3.5) from supine (64.9 ± 5.4) to standing (61.4 ± 5.6) in the whole cohort and was similar between volunteers and symptomatic cohort (−4° ± 4° vs. −4° ± 3°; p = 0.232) (Fig. 2). Men and women had ΔSFP of 3.8° ± 2.7° and 4.2° ± 2.6°, respectively (p = 0.053).
2. Acetabular morphology between supine and standing positions
  Parameter measurements demonstrated comparable changes in both groups from supine to standing position, except for ΔLCEA, with intergroup differences that remained below threshold of clinical relevance. In both groups, ΔAWI decreased from supine to standing (volunteers: −0.10 ± 0.08; p < 0.001 and symptomatic: −0.09 ± 0.07; p < 0.001) and ΔPWI increased (volunteers: 0.09 ± 0.08; p < 0.001 and symptomatic: 0.09 ± 0.07; p < 0.001), leading to a reduction in the ΔAWI/PWI ratio (volunteers: 0.15 ± 0.12; p < 0.001 and symptomatic: 0.13 ± 0.11; p < 0.001). Similarly, the prevalence of retroversion signs (COS, ISS, PWS) decreased from supine to standing.
  Accordingly, the prevalence of “anterior dysplasia” increased when moving from supine to standing (volunteers: 7 [7%] vs. 19 [18%]; symptomatic: 14 [3%] vs. 43 [10%]), whereas the prevalence of retroversion decreased (volunteers: 9 [9%] vs. 4 [4%]; symptomatic: 28 [7%] vs. 4 [1%]) (Table IV).
3. Change according to distinct acetabular morphologies
  Patients with signs of posterior dysplasia (COS, ISS, PWI < 0.8) in supine position had greater change in acetabular parameters compared with standing position, whereas those with anterior deficiency (AWI < 0.3) in supine position had smaller changes when compared with standing position (Table V). In patients with anterior dysplasia, ΔSFP was less compared with other patients (1.5° ± 2.5° vs. 3.7° ± 2.9°; p = 0.004), resulting in smaller ΔAWI (−0.03 ± 0.08 vs. −0.09 ± 0.07; p = 0.002) and ΔPWI (0.02 ± 0.06 vs. 0.09 ± 0.07; p = 0.001). In the contrary, in patients with posterior dysplasia, ΔSFP was greater (4.5° ± 2.7° vs. 3.6° ± 2.9°; p = 0.128), leading to larger ΔAWI (−0.12 ± 0.08 vs. −0.09 ± 0.07; p = 0.029) and ΔPWI (0.13 ± 0.08 vs. 0.08 ± 0.07; p = 0.001). Among patients with lateral dysplasia, ΔSFP did not differ significantly from the rest of the cohort (−3.6 ± 2.8 vs. −3.7 ± 2.9; p = 0.883). In the multivariate analysis, factors influencing absolute SFP change were found to be gender, anterior dysplasia, and posterior dysplasia (Table VI).

## Discussion

Understanding posture-related changes in pelvic position and their reciprocal effect on acetabular orientation is an important aspect of the diagnostic workup of patients with hip pain^10-12,32^. In the standing position, the acetabulum is assessed under joint-loading conditions, which is of relevance as pain upon loading and weight-bearing is common amongst patients presenting to clinic^9,33^. Nevertheless, normative acetabular values have to-date mostly been characterized on supine radiographs^1,5,27,34,35^. This study demonstrated that transitioning from supine to standing position was associated with a small increase in PT, resulting in greater posterior-acetabular and reduced anterior-acetabular coverage. The magnitude of these changes varied considerably among individuals, with similar magnitude of change and variability seen between asymptomatic volunteers and symptomatic patients, indicating that the presence of symptoms contributes little to this physiological adaptation upon standing. However, the magnitude of change was associated with the underlying acetabular morphology, regardless of symptoms. Upon standing, individuals adjusted their tilt accordingly (range of 22°), likely to optimize femoral head coverage by the acetabulum. Individuals with posterior-lateral acetabular deficiency tilted pelvis more, whilst those with isolated deficiency anteriorly, minimized adjustment. Thus, one could argue that if one AP pelvic radiograph was to be used, a standing assessment would be of preference because (1) it provides information upon loading/physiological conditions; (2) accounts for native compensatory mechanisms, which vary between individuals; (3) does not yield lateral coverage differences relative to supine assessment; (4) are associated with reduced false positive retroversion signs due to anterior inferior iliac spine (AIIS) morphology, compared with supine assessments; and (5) can be reproduced during surgical procedures (e.g., PAO) by beam adjustment considering SFP and pelvic appearances.

Both asymptomatic volunteers and symptomatic patients on average increased PT by 4° upon standing but showed considerable variability of change (19°-22°). These findings are in line with reports of patients with hip arthritis and postarthroplasty (range: 25°-30°)^15,36^, emphasizing importance of considering this for patient management. PT change resulted in minimal changes in acetabular parameters that assess lateral femoral head coverage (LCEA and AI), as previously reported in patients^26,32,33,37^ and volunteers^38^. By contrast, anterior and posterior acetabular coverage parameters were influenced by tilt change; upon standing AWI decreased while PWI increased, resulting in reduced AWI/PWI ratio and acetabular retroversion signs, in line with reports in dysplasia^11^, and retroversion^6,16^. Importantly, the changes in acetabular parameters between postures were similar in volunteers and symptomatic patients, suggesting that these adaptations are driven more by morphological characteristics rather than symptoms.

Patients with lateral undercoverage showed PT changes like those observed in asymptomatic individuals. This is probably due to the inability of PT change to compensate for lateral deficiency during the postural transition. On the contrary, patients with anterior wall deficiency demonstrated minimal posterior PT when transitioning from supine to standing. Conversely, patients with acetabular retroversion exhibited a marked increase in posterior PT when standing. In addition, women illustrated greater ability to change tilt in line with findings by others^11,26^. We hypothesize that these observations uncouple compensatory mechanisms to improve load bearing of the hip upon standing^39-41^. Furthermore, improvement in coverage over that postero-lateral area has been shown to be associated with outcome following osteotomy^42^.

Standing radiographs account for functional adaptations and reveal coverage deficits that one is unable to fully compensate for, providing a more accurate evaluation of true structural insufficiency. Furthermore, standing radiographs minimize false negatives due to AIIS morphology and improved specificity in detecting acetabular retroversion^6^. Thus, with the recommended utilization of CT for diagnosis and management of hip pathology, enabling accurate quantification of acetabular coverage (sector angles, subtended angles and LCEA in various positions around the acetabular clockface), the use of standing radiographs is to be recommended.

This study has several limitations. First, acetabular assessment and change in tilt were assessed on AP pelvic radiographs, which can be influenced by x-ray beam centering. While stereographic imaging (EOS) or CT scan in both supine and standing positions would eliminate this limitation, such modalities are not widely available, and EOS cannot be used in the supine position. Nonetheless, this study reflects pragmatic clinical conditions and prior cadaveric and modelling studies have shown strong correlations between PT and acetabular appearance, suggesting that beam centering effects are minimal^43,44^. Second, this study did not assess the relationship between PT compensation and surgery performed or hip functional outcomes, which would have provided insight into the clinical relevance of such adaptive mechanisms. However, such analysis was beyond the scope of this project and can be addressed in future investigations. Thirdly, this study did not account for pelvic movements in axial plane (i.e., rotation) that might influence acetabular orientation further, which was out of scope as it is not reliable parameter to quantify radiographically.

## Conclusion

On average PT increases from supine to standing by 4° ± 4°. This PT change optimizes loading pattern, and its magnitude and direction are associated with underlying acetabular morphology rather than presence of symptoms. These findings highlight the importance of standing radiographs for a functionally relevant assessment of acetabular coverage.

## Appendix

Supporting material provided by the authors is posted with the online version of this article as a data supplement at jbjs.org (http://links.lww.com/JBJSOA/B254). This content was not copy edited or verified by JBJS
